# Do Barrier Films Impact Long-Term Skin Toxicity following Whole-Breast Irradiation? Objective Follow-Up of Two Randomised Trials

**DOI:** 10.3390/jcm12227195

**Published:** 2023-11-20

**Authors:** Cas Stefaan Dejonckheere, Kira Lindner, Anne Bachmann, Alina Abramian, Katharina Layer, Teresa Anzböck, Julian Philipp Layer, Gustavo Renato Sarria, Davide Scafa, David Koch, Christina Leitzen, Christina Kaiser, Andree Faridi, Leonard Christopher Schmeel

**Affiliations:** 1 Department of Radiation Oncology, University Hospital Bonn, 53127 Bonn, Germany; katharina.layer@ukbonn.de (K.L.); christina.leitzen@ukbonn.de (C.L.); christopher.schmeel@ukbonn.de (L.C.S.); 2 Department of Gynaecology, Division of Senology, University Hospital Bonn, 53127 Bonn, Germany; 3 Department of Gynaecology, Division of Gynaecological Oncology, University Hospital Bonn, 53127 Bonn, Germany; 4 Institute of Experimental Oncology, University Hospital Bonn, 53127 Bonn, Germany

**Keywords:** hydrofilm, breast cancer, radiotherapy, pigmentation changes, skin fibrosis, objective assessment

## Abstract

**Purpose:** Hydrofilm, a polyurethane-based barrier film, can be used to prevent acute radiation dermatitis (RD) in adjuvant whole-breast irradiation (WBI) for breast cancer. This cost-effective prophylactic measure is currently being recommended to a growing number of patients, yet long-term safety data and its impact on late radiation-induced skin toxicity such as pigmentation changes and fibrosis have not been investigated. **Methods:** We objectively evaluated patients who were previously enrolled in either of two intrapatient-randomised (lateral versus medial breast halve) controlled trials on the use of Hydrofilm for RD prevention (DRKS00029665; registered on 19 July 2022). **Results:** Sixty-two patients (47.7% of the initial combined sample size) provided consent for this post-hoc examination, with a median follow-up time (range) of 58 (37–73) months. Following WBI, there was a significant increase in yellow skin tones of the entire breast when compared to baseline measurements before WBI (*p* < 0.001) and a significant increase of cutis, subcutis, and oedema thickness (*p* < 0.001, *p* < 0.001, and *p* = 0.004, respectively). At follow-up, there were no significant differences in either pigmentation changes or skin fibrosis between the Hydrofilm and standard of care breast halves. **Conclusion:** These data suggest that Hydrofilm can be safely used in the context of acute RD prevention, without affecting late side effects, supporting its widespread use.

## 1. Introduction

The most common oncological diagnosis worldwide remains early breast cancer [1]. In about 70% of cases, surgical therapy is followed by adjuvant radiation treatment to improve local tumour control and survival [2,3]. The most common side effect of whole-breast irradiation (WBI) is radiation dermatitis (RD), which occurs in up to 85% of patients and is characterised by erythema, pruritus, pain, and dry or moist desquamation [4,5]. Ongoing improvements in radiation treatment techniques (e.g., intensity-modulated radiation therapy [IMRT]) and the exploration of new fractionation regimens (e.g., hypofractionation) have led to a reduction in the breast skin dose, which in turn results in fewer and milder RD [4,6]. Severe cases necessitating radiation treatment interruption have thus become rare; however, even mild symptoms can impact a patient’s quality of life and self-image [7,8]. Even though continuous research efforts are being made, there is a lack of potent preventative and therapeutic options, leading to substantial variation in RD management amongst practitioners [9,10,11].

Hydrofilm^®^, a polyurethane-based barrier film, has been shown to majorly reduce the clinical appearance and patient-experienced symptoms of RD across multiple trials [12,13,14,15,16]. Based on these data, recently published international guidelines recommend its use for RD prevention in the context of adjuvant WBI [17]. Hydrofilm provides a mechanical barrier that protects the underlying radiation-damaged skin layers from additional friction or maceration and facilitates repair. It is a transparent and breathable film with an insignificant bolus effect, which is why it can be left on the skin for the entirety of the radiation treatment, promoting patient comfort. Side effects due to its application are mild and mostly self-limiting.

Apart from acute radiation-induced toxicity such as RD, breast cancer survivors might also be affected by late toxicities such as telangiectasia, pigmentation changes, or (sub)cutaneous fibrosis or induration [18]. These side effects can be irreversible and negatively impact breast cosmesis, which may result in an ongoing impairment of quality of life [19,20,21]. Evidence on the prevention and treatment of such late cutaneous toxicities is clearly limited. Reasons are manifold and include poor correlation between clinician- and patient-reported outcomes, limited availability of non-invasive objective assessment methods, and delayed diagnosis due to its late onset, inadvertently burdening numerous breast cancer patients [22,23].

Several high-quality trials have investigated the use and benefits of Hydrofilm and other barrier films for the prevention of acute radiation-induced skin toxicity; however, no data on its long-term impact on the irradiated skin exist. Here, we report the long-term follow-up of two previously published intrapatient-randomised controlled trials on the use of Hydrofilm for acute RD. We sought to determine the impact of Hydrofilm on late cutaneous toxicity following WBI, using validated objective assessment methods.

## 2. Materials and Methods

### 2.1. Patient Selection

Patients were drawn from two previously published prospective investigator-initiated intrapatient-randomised controlled trials on the use of Hydrofilm for the prevention of acute RD in the context of WBI for breast cancer [12,13]. Inclusion criteria for these initial trials were age > 18 years, breast-conserving surgery for breast cancer, and a fractionation regimen of 50 Gy in 25 fractions (conventional fractionation) or 40.05 Gy in 15 fractions (moderate hypofractionation). A conventionally fractionated sequential tumour bed boost of 16 Gy in 8 fractions was allowed. In order to minimise confounding factors, both trials defined similar exclusion criteria: Neoadjuvant or concomitant chemotherapy, mastectomy, reconstruction with breast implant, history of breast irradiation, metastatic disease, active smoking, active dermatitis, current treatment with oral or topical corticosteroids (known to suppress RD development), alternative fractionation regimens, and refusal to participate. These criteria resulted in two homogeneous patient collectives.

All patients who completed either of the initial trials per protocol were reassessed for inclusion in this post-hoc analysis. The following exclusion criteria were defined: Reconstruction with breast implant, recurrent or metastatic disease, active dermatitis, and current use of oral or topical corticosteroids. Eligible patients were contacted by telephone and invited to participate. All assessments were completed between September 2022 and February 2023, after obtaining a written informed consent from all participants. This study was conducted in accordance with the Declaration of Helsinki and approved by the Institutional Review Board of the University of Bonn, Germany (184/22).

### 2.2. Radiation Protocol

All patients previously underwent adjuvant WBI. Those < 50 years or those with risk factors (≥pT2, HER2 positive, triple-negative, or poor cell differentiation, as per German national guidelines), regardless of age, received a sequential boost to the tumour bed. For both trials, the treatment technique used was 6 MV sliding window IMRT or hybrid 6 and 10 MV volumetric modulated (partial) arc therapy (VMAT). The International Commission on Radiation Units and Measurements (ICRU) recommendations for dose limits of 95–107% were followed. If feasible, left-sided WBI was performed in deep inspiration breath-hold (DIBH), with all patients in a supine position on a breast board. Patients were treated on a TrueBeam STx (Varian Medical Systems, Palo Alto, CA, USA) linear accelerator.

### 2.3. Intervention

In both initial trials, patients acted as their own control: The irradiated breast was divided into a lateral and medial compartment, and these halves were randomised to receive either Hydrofilm or standard of care. Hydrofilm (Paul Hartmann, Heidenheim, Germany) is a sterile polyurethane film with a hypoallergenic polyacrylate adhesive. Phantom studies revealed a clinically insignificant skin dose build-up (<0.1%) with Hydrofilm, which is why it can remain on the skin from the first day of radiation treatment until completion. Institutional standard of skin care consisted of urea lotion (UreaRepair PLUS 5%, Eucerin, Beiersdorf, Hamburg, Germany) twice daily applied from the first day of radiation treatment until completion. Topical corticosteroids were prescribed to patients with grade ≥ 2 RD, moist desquamation, and/or intense pain, until symptoms resolved.

### 2.4. Patient Evaluation

Biometric data and patient characteristics were collected. As clinician- and patient-reported outcomes are difficult to assess for each breast halve separately, especially regarding late skin toxicity, previously validated objective skin assessment methods were used.

(a) Evaluation of Pigmentation Changes with Spectrophotometry

**Figure 1:**
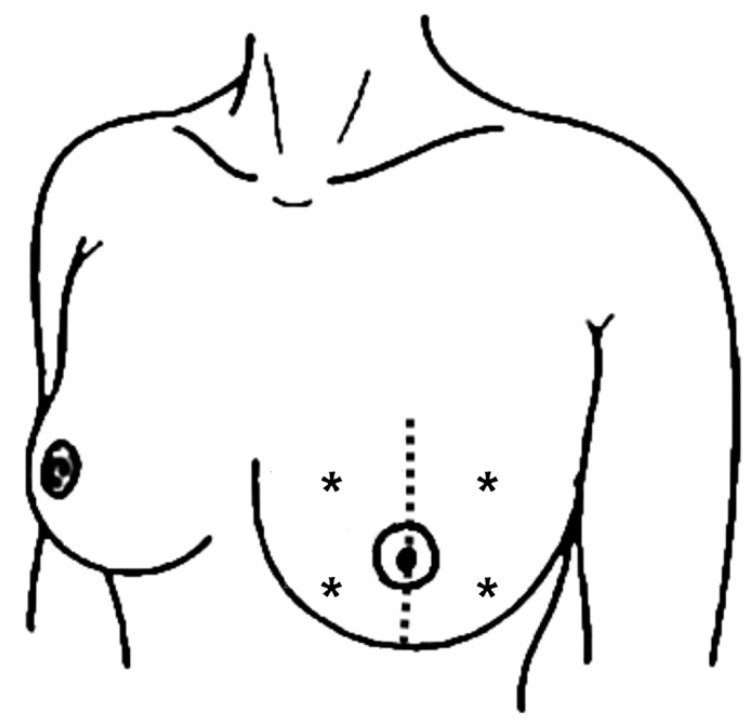
The four stars indicate the location of the spectrophotometric readings and ultrasound measurements at follow-up.

Four erythema readings were performed on the previously irradiated breast (two lateral and two medial) with a reflectance spectrophotometer (CR-10 Plus, Konica Minolta, Tokyo, Japan) (Figure 1). These automatically performed measurements are based on the system of tristimulus values of the Commission Internationale de l’Éclairage (CIE). They describe a measured colour in three coordinates using the L*a*b* system. This technique has been previously validated in the context of acute RD: Lower L* values describe darker skin (hyperpigmentation) and higher a* values indicate redness (RD), whereas b* values describe the position of a colour on a scale from blue to yellow (of secondary importance in the acute setting) [24,25]. As this technique was also used in both initial trials, baseline values (before initiation of radiation treatment) for each patient and for both breast halves separately were available (there were no relevant differences in baseline measurements between breast halves in both patient groups). The clinician who performed the automated measurements was blinded to the intrapatient randomisation (i.e., which breast halve had previously been covered with Hydrofilm).

(b) Evaluation of Skin Fibrosis with Ultrasound

To assess the potential impact of Hydrofilm on tissue fibrosis, skin thickness was measured using ultrasound. This method previously proved reliable and valid in the context of late radiation-induced toxicity [26]. All breast examinations were performed in B-mode, using 4–13 MHz ML6-15-D linear array probes on a Voluson E10 (GE Healthcare, Solingen, Germany). Patients were in a supine position, and the axis of the ultrasound probe was held perpendicular to the skin surface, applying minimal pressure. To guarantee proper coupling, a thin layer of transmission gel was used. The thickness of the cutis (epidermis and dermis) and subcutis were measured in each of the breast quadrants (1, 4, 8, and 10 o’clock) (Figure 1). Anatomically, the transition between cutis and subcutis can be determined very precisely, as the epidermal part of the cutis consists mainly of keratinocytes, whereas its dermal part and the subcutis are connective and adipose tissue, reflected by a different image morphology on the ultrasound image. In the case where subcutaneous oedema was present, this was documented and quantified with ultrasound as well. Measurements at follow-up were compared with baseline values (before initiation of radiation treatment). To avoid interobserver variability, all measurements were performed by a single senior breast surgeon with certified expertise in breast ultrasound, who was also blinded to the intrapatient randomisation, to further reduce bias.

### 2.5. Statistical Analysis

Mean, median, standard deviation (SD), and range were calculated for all applicable clinical data. To compare objective outcomes (spectrophotometry and ultrasound) to baseline values for the same patient, a paired *t*-test was performed. To compare current objective outcomes between Hydrofilm and control halves, the unpaired two-samples *t*-test was used. The statistical significance level was defined as *p* < 0.05. SPSS Statistics version 27 (IBM, Armonk, NY, USA) was used to perform the analyses.

## 3. Results

### 3.1. Patient Characteristics

Of 130 patients successfully completing either of the initial trials, 62 (47.7%) consented to the follow-up examination and yielded data for this analysis (Table 1). Median age (range) was 59 (37–81) years, whereas median follow-up time (range) was 58 (37–73) months after WBI. Approximately 98.4% of patients were female, and 95.2% were Caucasian. Further patient and radiation treatment characteristics are presented in Table 2.

### 3.2. Objective Outcomes

(a) Pigmentation Changes (Spectrophotometry)

Baseline spectrophotometric data (before irradiation) were compared with the means of four ipsilateral measurements at follow-up. A slight decrease in L* value and an increase in a* value (indicating more black and more red, respectively) were observed. These differences, however, were not significant. On the other hand, a significant increase in b* value (indicating more yellow) was observed (*p* < 0.001). Results are summarised in Table 3.

At follow-up, both halves of the irradiated breast (Hydrofilm versus standard of care) were compared. No significant differences in either L*, a*, or b* values were observed. Results are summarised in Table 4.

(b) Skin Fibrosis (Ultrasound)

Baseline ultrasonographic measurements were compared with the means of four ipsilateral measurements at follow-up. Overall, there was a significant increase in skin thickness with a doubling of cutis, subcutis, and subcutaneous oedema dimensions following irradiation. Table 5 summarises these results.

At follow-up, both halves of the irradiated breast (Hydrofilm versus standard of care) were compared. No significant differences in either cutis, subcutis, or subcutaneous oedema thickness were observed. Results are summarised in Table 6.

## 4. Discussion

Barrier films such as Hydrofilm prove useful in the prevention of acute RD during WBI for breast cancer. They have been shown to improve clinician- and patient-reported outcomes and subsequent quality of life. An increased interest in this cost-effective prophylactic measure, reflected by a recent surge of publications on this topic, prompts the need for an assessment of the impact of barrier films on late skin toxicities. Here, we report the long-term follow-up of two previously published intrapatient-randomised controlled trials to assess potential differences in pigmentation changes and fibrosis between breast skin compartments that have been treated with Hydrofilm or standard of care and to establish the long-term safety of barrier films.

Overall, a slight difference in skin pigmentation (more yellow tones) and a marked increase in overall skin thickness, reflecting fibrosis, were observed when comparing irradiated breasts to baseline measurements before WBI initiation. These objective results corroborate previous (mainly clinician-reported) findings of increased skin fibrosis following WBI with contemporary radiation techniques [20,21,27].

However, when comparing pigmentation and skin thickness between Hydrofilm and standard of care breast halves, no significant differences were observed. Hydrofilm application during WBI thus does not impact late cutaneous toxicity rates, neither positively nor negatively.

Other barrier films with a similar mechanism of action to Hydrofilm are available and are being used to prevent acute RD in adjuvant WBI. Mepitel^®^ film, a commonly used silicone-based barrier film, results in a similar improvement of both clinician- and patient-reported outcomes [28,29,30]. Due to its very similar properties and mode of use, we reason that its impact on late toxicity may be similar to that of the here investigated Hydrofilm. The evidence on other barrier films, dressings, and creams, as well as film-forming gels, in the context of acute RD prevention is limited [14]. Furthermore, their impact on late toxicity has not been investigated.

An increasing number of other interventions for acute RD prevention have been or are being investigated, with varying results. Due to insufficient and sometimes even conflicting evidence, a recommendation can currently only be made for a handful of interventions [17]. Of all natural agents, only oral enzymes and olive oil have proved useful so far [31,32]. Since the publication of the Multinational Association of Supportive Care in Cancer (MASCC) guideline, other topical agents such as epigallocatechin-3-gallate have been investigated successfully [33].

Topical corticosteroids (mometasone, betamethasone) are effective in reducing RD-related symptoms such as pain, burning, or itching; however, their widespread and prolonged use remains limited due to the associated side effect profile [34,35,36]. Care should also be taken if moist desquamation is present, as topical corticosteroids might then delay wound healing or promote infection.

Lastly, medical devices such as photobiomodulation therapy have also proved useful, especially in the context of breast cancer [37]. Other devices for acute RD prevention are currently being investigated [38,39,40].

Even though many trials have investigated measures to prevent acute RD, none have considered the impact of these interventions on late cutaneous toxicities. Furthermore, there is a general lack of studies exploring preventative or therapeutic interventions for late radiation-induced toxicity. Additional research on these topics is desirable to prevent adverse cosmetic outcomes and improve quality of life of (breast) cancer survivors. Another valuable approach towards reducing acute side effects are advances in radiation treatment techniques and patient positioning [41]. The impact of such developments on late toxicity should also be investigated in the future.

Our study is not without limitations. First, only half of the initial study population could be recruited for this follow-up examination (almost 40% of patients could either not be reached or declined participation), resulting in a relatively small sample size. Therefore, subgroup analyses (e.g., based on fractionation regimen or Fitzpatrick skin type, factors known to influence radiation toxicity) were not performed, and it is currently unknown if certain subgroups might have benefited from Hydrofilm application in terms of late skin toxicity, which should be assessed in future prospective trials. Furthermore, due to the intrapatient-randomised design of both initial trials, an assessment of clinician- and patient-reported late toxicities was not possible, as this requires an evaluation of the entire irradiated breast. If each patient acts as their own control, confounding can be minimised, which omits the need for stratification based on other factors known to influence the RD risk (e.g., breast size, Fitzpatrick skin type, fractionation regimen), which is why this design was chosen in the first place. Furthermore, external factors influencing the development of pigmentation changes and/or skin fibrosis (e.g., skin care routine, sun exposure) can be disregarded due to intrapatient randomisation. The use of validated objective assessment methods in the current trial could also partially bypass this. Long-term follow-up of similar trials with a physical control group who did not receive barrier film will further elucidate this in the future [30].

## 5. Conclusions

To the best of our knowledge, we provide the first comprehensive assessment with a mature follow-up of the impact of Hydrofilm on late skin toxicity following adjuvant WBI for breast cancer. Even though no improvements in the rate of pigmentation changes or skin fibrosis were observed, the data suggest that Hydrofilm can be safely used in the context of acute RD prevention, without affecting said late radiation-induced side effects. The data thus support the widespread use of barrier films for the prevention of acute RD in WBI.

## Figures and Tables

**Table 1:** Overview of patient selection and inclusion.

	Schmeel et al. 2018 (September 2016–September 2017) [12]	Schmeel et al. 2019 (March 2018–June 2019) [13]	Total
Enrolled	62	80	142
Included	56	74	130
deceased			−8
recurrent or metastatic disease			−10
refusal to participate			−22
not reached			−28
Included	33	29	62

**Table 2:** Patient and radiation treatment characteristics (*n* = 62).

	Median (Range)
Age in years	59 (37–81)
Follow-up time in months	58 (37–73)
	***n* (%)**
Female	61 (98.4)
Caucasian	59 (95.2)
Diabetes mellitus	1 (1.6)
Active smoking	11 (17.7)
Fitzpatrick skin type	
I	11 (17.7)
II	44 (71.0)
III	6 (9.7)
V	1 (1.6)
Fractionation regimen	
50 Gy in 25 fractions	33 (53.2)
40.05 Gy in 15 fractions	29 (46.8)
Sequential boost	31 (50.0)
Hydrofilm halve	
lateral	30 (48.4)
medial	32 (51.6)

**Table 3:** Spectrophotometric comparison of baseline breast colour and combined ipsilateral values at follow-up (*n* = 558 readings).

	Baseline	Follow-Up	Δ	*p*
Mean L*	69.6	69.3	−0.3	0.191
Mean a*	6.5	6.6	+0.1	0.326
Mean b*	14.1	14.8	+0.7	<0.001

**Table 4:** Spectrophotometric comparison of Hydrofilm and control breast halves at follow-up (*n* = 248 readings).

	Hydrofilm	Control	Δ	*p*
Mean L*	69.2	69.1	+0.1	0.911
Mean a*	6.6	6.7	−0.1	0.786
Mean b*	14.9	15.0	−0.1	0.811

**Table 5:** Ultrasonographic comparison of baseline breast skin thickness and combined ipsilateral values at follow-up (*n* = 496 readings).

	Baseline	Follow-Up	Δ	*p*
Cutis (mm)	1.9	3.8	+1.9	<0.001
Subcutis (mm)	2.7	5.5	+2.8	<0.001
Oedema (mm)	2.3	4.5	+2.2	0.004

**Table 6:** Ultrasonographic comparison of Hydrofilm and control breast halves at follow-up (*n* = 248 measurements).

	Hydrofilm	Control	Δ	*p*
Cutis (mm)	3.7	3.9	−0.2	0.525
Subcutis (mm)	5.5	5.5	0	0.918
Oedema (mm)	3.8	5.3	−1.5	0.521

## Data Availability

Research data are available upon reasonable request.
